# Prediction Equation for Physical Activity Energy Expenditure in 11–13-Year-Old Sri Lankan Children

**DOI:** 10.3390/nu15040906

**Published:** 2023-02-10

**Authors:** Prasangi Dabare, Pujitha Wickramasinghe, Indu Waidyatilaka, Sarita Devi, Anura V. Kurpad, Dulani Samaranayake, Maduka de Lanerolle-Dias, Rajitha Wickremasinghe, Andrew P. Hills, Pulani Lanerolle

**Affiliations:** 1 Department of Physiotherapy, Faculty of Allied Health Sciences, General Sir John Kotelawala Defence University, Dehiwala-Mount Lavinia 10390, Sri Lanka; 2 Department of Pediatrics, Faculty of Medicine, University of Colombo, Colombo 00800, Sri Lanka; 3 Department of Biochemistry and Molecular Biology, Faculty of Medicine, University of Colombo, Colombo 00800, Sri Lanka; 4 Division of Nutrition, St. John’s Research Institute, Sarjapur Road, Bengaluru 560034, India; 5 Department of Physiology, St. John’s Medical College, Sarjapur Road, Bengaluru 560034, India; 6 Department of Community Medicine, Faculty of Medicine, University of Colombo, Colombo 00800, Sri Lanka; 7 Department of Public Health, Faculty of Medicine, University of Kelaniya, Kelaniya 11010, Sri Lanka; 8 School of Health Sciences, College of Health and Medicine, University of Tasmania, Hobart, TAS 7005, Australia

**Keywords:** accelerometers, physical activity energy expenditure, children, validation, stable isotopes

## Abstract

This study aimed to develop a regression equation to predict physical activity energy expenditure (PAEE) using accelerometry. Children aged 11–13 years were recruited and randomly assigned to validation (*n* = 54) and cross-validation (*n* = 25) groups. The doubly labelled water (DLW) technique was used to assess energy expenditure and accelerometers were worn by participants across the same period. A preliminary equation was developed using stepwise multiple regression analysis with sex, height, weight, body mass index, fat-free mass, fat mass and counts per minute (CPM) as independent variables. Goodness-of-fit statistics were used to select the best prediction variables. The PRESS (predicted residual error sum of squares) statistical method was used to validate the final prediction equation. The preliminary equation was cross-validated on an independent group and no significant (*p* > 0.05) difference was observed in the PAEE estimated from the two methods. Independent variables of the final prediction equation (PAEE = [0.001CPM] − 0.112) accounted for 70.6% of the variance. The new equation developed to predict PAEE from accelerometry was found to be valid for use in Sri Lankan children.

## 1. Introduction

The importance of physical activity in maintaining and promoting overall health and well-being, as well as reducing the risk of non-communicable diseases (NCDs) and obesity is well known [1]. There is also evidence of the detrimental effects of insufficient physical activity and high levels of sedentary behavior on physiological, psychological and social health among all age groups, including children and adolescents [1]. Physical activity is a key contributor to total energy expenditure (TEE) and is essential in maintaining an equilibrium between energy intake and energy expenditure [2]. Over time, there has been increased interest in the assessment of TEE and its components including physical activity energy expenditure (PAEE) while studying energy balance among children. TEE comprises basal metabolic rate (BMR), PAEE and the thermic effect of food (TEF). Typically, with measured or estimated BMR, and assuming that TEF represents approximately 10% of TEE, PAEE is predicted as follows: PAEE = 0.9 TEE-BMR [3,4,5]. The doubly labelled water (DLW) technique is the gold standard for the assessment of TEE under free-living conditions [2]. However, the use of the DLW technique in field settings is limited due to the relatively high cost of purchase and analysis of stable isotopes [6]. Therefore, other less invasive and more cost-effective subjective methods, such as questionnaires, diaries/activity logs and objective methods, including activity monitors, are commonly used. As the use of physical activity questionnaires and logs/diaries among children and adolescents is particularly challenging due to recall and reporting biases [7], objective methods, including accelerometers, are in widespread use to assess physical activity and energy expenditure in young people [8,9,10].

Accelerometers provide information regarding activity characteristics, including intensity (light, moderate, vigorous), frequency, sedentary behavior and energy expenditure [11,12]. Advantages of accelerometers include being less invasive, convenient to handle lightweight with minimum disturbance to daily activities and the ability to record data continuously for an extended period of time [13]. Different types of accelerometers have been validated in different populations [3,4,12]. However, at present, there are no published regression equations using accelerometry to predict free-living PAEE in Sri Lankan children. Eight existing prediction equations were cross-validated among 79 Sri Lankan adolescents (41 boys) aged 11–13 years and all equations demonstrated significant errors in estimating energy expenditure [14]. Therefore, the current study aimed to develop and validate a regression equation to predict PAEE using accelerometry in Sri Lankan adolescents aged 11–13 years.

## 2. Materials and Methods

### 2.1. Study Participants, Selection and Study Design

A purposive sample of 96 adolescents (49 boys) aged 11–13 years attending two schools in Colombo, Sri Lanka, were recruited after obtaining informed written consent from parents/guardians and assent from children. Sixteen children were consecutively recruited from each age category (11, 12 and 13 years), from each school to represent the national distribution of nutritional status [15]. The study was conducted according to the Declaration of Helsinki and was approved by the Ethics Review Committee of the Faculty of Medicine, University of Colombo, Sri Lanka (EC/16/192). A detailed study design and recruitment procedures are published elsewhere [14].

### 2.2. Measurements

#### 2.2.1. Anthropometric Measures

The weight of participants was measured to the nearest 0.1 kg using a calibrated electronic scale (Seca 803^®^ by SECA GmbH & Co., Hamburg, Germany). Height was measured to the nearest 0.1 cm using a stadiometer (Seca 225^®^ by SECA GmbH & Co., Hamburg, Germany). All measurements were performed by a single trained researcher in duplicate using the same equipment and following the standard procedures according to the International Society for the Advancement of Kinanthropometry [16]. Body mass index (BMI) was calculated as weight divided by height squared (kg/m^2^).

#### 2.2.2. Total Energy Expenditure (TEE)

TEE was assessed using the DLW technique. The standard procedures in the DLW technique, including dose preparation, dose storage, administration, sample collection and sample storage plus analysis were carried out according to the International Atomic Energy Agency (IAEA) protocol [2]. The DLW dose was a weighed mixture of 0.12 g.kg^−1^ body water of 99.8% ^2^H_2_O and 1.8 g.kg^−1^ body water of 10% H_2_^18^O (Sigma-Aldrich Co., St. Louis, MO, USA) [2]. Four large stock dose bottles (1 L each) were prepared such that the dose was sufficient for the total number of participants and stored in glass bottles with a screw cap at 4 °C. The individual doses were weighed to the nearest 0.0001 g using an analytical weighing balance. Doses were stored in pre-labelled leak-proof bottles at 4 °C, until the dosing date.

Data collection was carried out in local schools. On the dosing day (Day 1), a baseline urine sample was collected before the dose administration, then the DLW dose was administered under the supervision of the investigator. Four hours following the administration of the dose, the first post-dose urine sample was collected. On day 10, the final urine sample was collected at the same time as the 4th-hour post-dose urine sample on day 1. All samples were stored at −20 °C prior to analysis. An isotope-ratio mass spectrometer (IRMS, Delta V Advantage, Thermo Scientific, Bremen, Germany) at the Mass Spectrometry Laboratory, St. John’s Research Institute, Bangalore, India was used to analyze the isotopic enrichments of ^2^H and ^18^O in the urine samples. The precision of ^2^H and ^18^O measurements was <1.0% and <0.08%, respectively.

Total body water (TBW) was first calculated using the isotopic enrichment and corrected for the non-aqueous hydrogen exchange [2]. Fat-free mass (FFM) was estimated from the corrected TBW by using age and a sex-specific hydration coefficient [17], and fat mass (FM) was then calculated by subtracting FFM from body weight. The Schoeller et al. [18] equation was used to calculate the rate of CO_2_ production, and assuming an average food quotient of 0.86, the total energy expenditure (TEE_DLW_) was calculated using the modified Weir equation [19]. Assuming 10% of TEE would be the thermic effect of food (TEF), the PAEE_DLW_ was calculated as 0.9 TEE_DLW_ − BMR [20]. BMR was calculated using the prediction equation of Schofield [21].

#### 2.2.3. Accelerometry

ActiGraph wGT3x-BT^®^ triaxial accelerometers (Pensacola, FL, USA) were used in this study to assess physical activity. Each participant wore the accelerometer on their waist during the same 10 days of the DLW protocol. Participants wore the device during waking hours and were instructed to remove it during sleep and all water-based activities such as swimming and bathing. All accelerometers were tested prior to distribution to participants and machines were initialized. The sampling frequency was set at 30 Hz which was identified as suitable for children of this age [22,23]. Additionally, a systematic review by Migueles and colleagues [24] stated that the majority (64%) of studies using the ActiGraph in children and adolescents used a sampling rate of 30 Hz. Accelerometer data were analyzed using the Actilife software^®^, version 6 (Pensacola, FL, USA). Durations of 60 min or more of continuous zeros, allowing for 2 min of non-zero intervals, were considered as non-wear time and were excluded from the analysis [9,25]. Days were considered valid if at least 600 min of wear time was noted during wake time [25]. Data were included in the analysis if the participants completed a minimum of three such valid weekdays and one valid weekend day [9,25]. The movement was recorded in 1 s epochs and integrated into 15 s epochs for analysis [9,25]. Total activity counts were divided by the valid registered time to calculate the total CPM (counts per minute) [3,9].

### 2.3. Statistical Analysis

A statistical computer package NCSS 2000 (Hintze JL, Kayswille, UT, USA) was used to analyze the data which is presented as mean and standard deviation (SD). The Kolmogorov–Smirnov test was used to assess the normality of data and parametric tests and nonparametric analyses were used where appropriate. The results reported here include a final sample of 79 adolescents (38 girls and 41 boys). One participant was excluded due to incomplete urine sample collection for the DLW protocol and another four participants since their post-dose urine sample enrichments were lower compared to the baseline enrichment for ^2^H and ^18^O as measured using IRMS. A further four participants were excluded as the minimum accelerometer wear-time was not completed. The results of another eight participants were removed from the analysis since those values were identified as outliers (>±3 standard deviations from the mean in each data column) [26].

The male and female participants were listed by age in ascending order and every third participant was recruited into the cross-validation group (*n* = 25) and the remainder to the validation group (*n* = 54) for the development and validation of the PAEE equation.

The PAEE prediction equation was developed using stepwise multiple regression analysis with PAEE_DLW_ as the dependent variable and age, sex, weight, height, FFM, FM and CPM as possible independent variables. Goodness-of-fit statistics was used to select the best prediction variables. Girls were coded as 0 and boys as 1 during all analyses. Variance inflation factor >10 was used to assess the multicollinearity of the independent variables [27]. The prediction regression model with the highest coefficient of determination (R^2^) and the lowest root mean squared error (RMSE) was selected as the best prediction equation. The preliminary equation’s predictability was assessed in children of the cross-validation group by comparing PAEE calculated using the preliminary equation with the PAEE assessed by the criterion method using the paired sample t-test. Pure error calculated from the cross-validation group was compared with the RMSE calculated from the validation group for the similarity between the two values indicating better predictability of the preliminary equation in the cross-validation group [28].

The final prediction equation was constructed after combining the validation and cross-validation groups (*n* = 79) and the PRESS (predicted residual error sum of squares) statistical method was used to validate the equation [27,28]. The degree of agreement between the PAEE from the new prediction equation (PAEE_Predicted_) and PAEE_DLW_ was assessed by the Bland–Altman approach of assessing agreement between methods [29] with the differences between the PAEE_DLW_ and PAEE_Predicted_ (*y*-axis) plotted against the average of PAEE_DLW_ and PAEE_Predicted_ (*x*-axis). The level of statistical significance was set at *p* < 0.05, for all tests.

## 3. Results

Basic demographic and anthropometric parameters of the validation and cross-validation groups are presented in Table 1. There was no statistically significant difference between the two groups for each sex using ANOVA (analysis of variance) at *p* < 0.05.

Stepwise multiple regression analysis was applied to the validation group (*n* = 54) using PAEE_DLW_ as the dependent variable and sex (girls were coded as 0 and boys as 1), height, weight, BMI, FFM, FM and CPM as possible independent variables. Equations were developed as sex-non-specific and sex-specific equations. Variance inflation factors of more than 10 were used to detect multicollinearity among the variables [27,28]. Based on goodness-of-fit statistics, only CPM was identified as the most reliable independent variable to predict PAEE in both sex-non-specific and sex-specific equations (*p* < 0.05) (Table 2).

Both sex, non-specific and sex-specific equations were constructed using CPM as the independent variable. The sex-specific equation did not significantly improve R^2^ and RMSE. Therefore, the sex non-specific equation was selected as the best-fitting preliminary equation to predict PAEE.

The predictability of the preliminary equation was evaluated in the cross-validation group. A significant correlation was observed between the PAEE predicted using the preliminary equation (R = 0.84) with the PAEE_DLW_ (*p* < 0.05). No significant difference was observed in the PAEE estimated using the gold standard method and the preliminary equation (0.39 ± 0.27 kcal/min vs. 0.36 ± 0.18 kcal/min, respectively (*p* > 0.05). The predictability of PAEE using the preliminary equation was also evaluated in the cross-validation group using pure error [27,28]. Good predictability identified as the RMSE value of the validation group (0.13 kcal/min) was similar to the pure error value of the cross-validation group (0.14 kcal/min).

The final prediction equation was constructed after combining both the validation and cross-validation groups (*n* = 79) and validated using the PRESS statistical method [27]. The value of the coefficient of determination of multiple regression analysis was close to the PRESS R2 (70.6% vs. 69.1%, respectively) showing a strong agreement. The PRESS statistic value of the total sample was compared with the pure error of the cross-validation group. The PRESS statistic was lower but comparable to the pure error value of the cross-validation group (0.13 kcal/min vs. 0.14 kcal/min).

**Figure 1:**
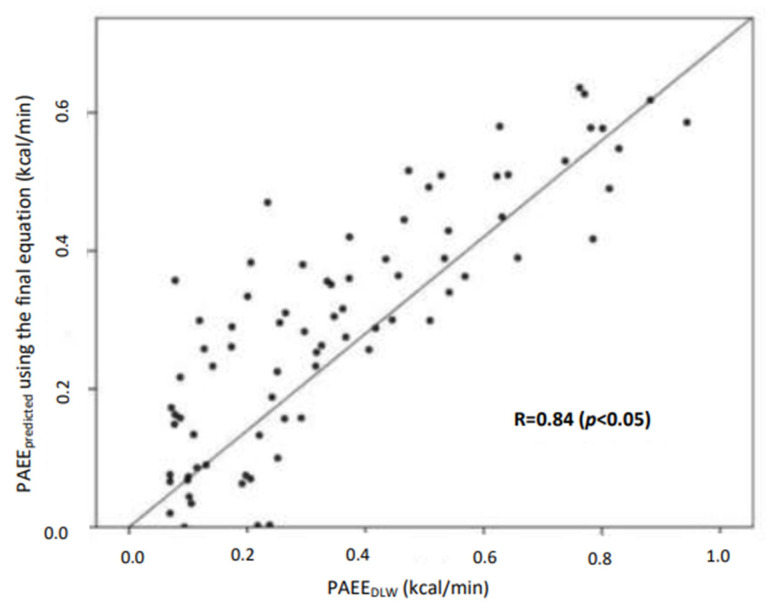
Regression line of the PAEE calculated using the final equation vs. the DLW technique.

Figure 1 presents the association between the PAEE estimated using the final prediction equation with the PAEE_DLW_. A significant correlation was observed between the estimated and measured PAEE (*p* < 0.05).

**Figure 2:**
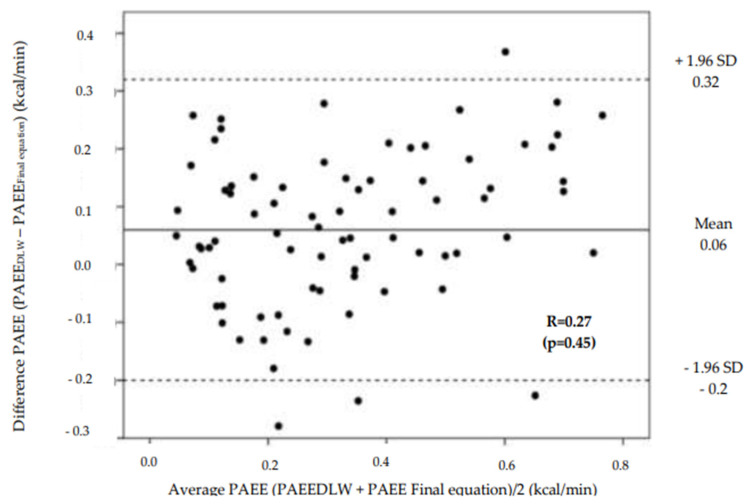
Bland–Altman plots showing bias and average between the PAEE calculated using the criterion method (DLW) and estimated using the final equation. The mid-line indicates the mean bias and the two dashed lines, the upper and lower agreement limits (mean bias ± 1.96 x standard deviations, 95%).

The distribution of the bias for PAEE calculated from the final prediction equation and assessed using DLW is plotted against the mean of the PAEE calculated from the final prediction equation and DLW according to the Bland–Altman technique (Figure 2). The mean bias of the final prediction equation was 0.06 ± 0.16 kcal/min with +0.32 kcal/min and −0.2 kcal/min being the upper and lower agreement limits (Figure 2).

## 4. Discussion

Most published accelerometer-based energy expenditure prediction equations have been developed and validated on Western adolescent populations. Hence, it is prudent to either cross-validate such equations on the desired study population or develop new equations to improve the accuracy of prediction. We have previously established that existing regression equations are unable to accurately estimate PAEE among the current study population [14]. Accordingly, we developed and validated a new regression equation to predict PAEE in Sri Lankan adolescents aged 11–13 years, using DLW as the criterion method for the first time in Sri Lanka.

Tri-axial accelerometers provide a more accurate estimate of physical activities and their ability to predict energy expenditure is reported to be higher compared to the uniaxial and biaxial devices since the acceleration that occurs in all three axes is captured [4,30,31,32,33]. This study used activity counts based on the vector magnitude which included activities occurring in all three axes. CPM recorded in our study showed a significantly high correlation (r = 0.81) with energy expenditure among the study population. Similarly, a good correlation (r = 0.76) was observed between CPM and energy expenditure in another study which used activity counts based on the vector magnitude [33]. In contrast, activity counts based on a single axis (vertical axis) have been shown to be comparatively poorly correlated with energy expenditure among adolescents [3,34]. Triaxial accelerometers are comparatively more subtle in capturing the torsional movements frequently involved in physical activities performed by children [35]. However, the majority of published energy expenditure equations have been constructed based on vertical activity counts and the use of tri-axial accelerometer counts to estimate energy expenditure using existing prediction equations is not known. Hence the current equation developed using the tri-axial accelerometer counts is a better predictor of PAEE in Sri Lankan children.

Applying stepwise multiple regression analysis on the validation group, resulted in only CPM being identified as a significant independent variable to predict PAEE. This is comparable to the equations developed by Puyau et al. [36] and Treuth et al. [34] which contained CPM as the only independent variable in energy expenditure estimates. Additionally, it is concluded that the relationship between energy expenditure and activity counts is independent of age and sex [33]. However, in addition to CPM, some other published regression equations included weight [33,35,37], age [38], sex [3] or both sex and age [39]. In our study, age was not observed to be a significant independent variable as we considered a narrow age range of 11–13 years. Although sex was considered, the sex-specific equations did not significantly improve the prediction of energy expenditure compared to the sex-non-specific equation.

The amount of variance (R^2^) explained by the final energy expenditure prediction equation of this study was lower than most of the previous studies [37,38,39] but better than that reported by Ekelund and colleagues [3]. The R^2^ of our study was comparable to that of the equation developed by Zhu and colleagues [33]. This may be explained by the fact that the equation developed by Zhu and colleagues is the only one developed and cross-validated among Asian adolescents that considered activity counts based on vector magnitude, similar to our study. Additionally, Zhu and colleagues [33] included both laboratory and free-living activities whereas most of the other studies used pre-planned laboratory-based and/or treadmill activities [35,37,38,39]. The high standard error of estimate (SEE) of 0.14 kcal/min of the final prediction equation of our study indicates a poor ability to predict energy expenditure at an individual level but not at a group level. However, the SEE we report is substantially lower than that in similar studies [33,36,37] indicating better prediction compared to existing regression equations.

We constructed the final prediction equation after combining the validation and cross-validation groups and PRESS statistic was used to validate the final equation. In the final prediction equation, the PRESS residual was lower than the mean bias in the cross-validation group indicating an overestimation of energy expenditure among our study population. Similar studies have not used PRESS statistics and there is no data to compare our final prediction equation with. Zhu and colleagues [33] cross-validated six different energy expenditure estimating equations among Chinese adolescents and the accuracy for prediction was compared by calculating the root mean square error (RMSE). The RMSE of all their equations were higher than that of this study, further emphasizing the inaccuracies of using those equations to predict the energy expenditure at an individual level. Therefore, the energy expenditure prediction equation of the current study was identified as more accurate than the existing equations to predict energy expenditure among Sri Lankan adolescents at a group level.

We calculated the TEE using the DLW technique with the modified Weir equation [19], assuming an average food quotient of 0.86. In Western countries where 30–35% of energy is provided from fat, a food quotient value of 0.85–0.86 is observed [2]. A few studies in Sri Lanka reporting on local dietary intake reported higher percentages of energy supplied by carbohydrates with less from fat and protein than Western diets which translate to food quotient values ranging from 0.91 to 0.94 [40,41]. Therefore, some error might be expected due to the assumptions centered around using 0.86. At the same time, it is noteworthy that the available local studies on dietary intake are not representative of the general Sri Lankan population and hence the applicability of the food quotient values from dietary intakes reported in these studies to our study population would be questionable. Additionally, according to Treuth and colleagues [42], accurate measurement of food quotient is not essential as TEE estimates can be expected to be in error by 1% for a 0.01-unit error in food quotient. There are some limitations of this study. We validated the equation against energy expenditure estimated using the DLW technique in which participants performed free-living activities for a period of 10 days. Most studies which developed equations have used various activities (walking, running, play activities, sweeping, jumping, etc.) and the commonly used criterion method has been indirect calorimetry. Therefore, the prediction ability of energy expenditure of different activities separately could not be evaluated using the current equation. We did not measure BMR as data collection was undertaken in schools. BMR was calculated using the Schofield [21] equation since there are no validated BMR prediction equations for Sri Lankan adolescents. Therefore, it is likely that some error may have been introduced in the energy expenditure calculations. We recommend that future studies include BMR assessment to improve the accuracy of the prediction of energy expenditure. Further, the criterion DLW technique cannot be used directly to assess PAEE. Direct measurements of PAEE, such as calorimetry, should be used in future studies to cross-validate the prediction equation we report here. We used waist-worn ActiGraph CPM to estimate energy expenditure. However, it may underestimate physical activity involving the upper limb [43,44]. Therefore, future studies should be undertaken to assess accelerometers on different body segments.

## 5. Conclusions

This study developed and validated a regression equation using accelerometry to estimate PAEE in 11–13-year-old Sri Lankan adolescents (PAEE = [0.001CPM] − 0.112). Further cross-validation studies will improve the generalizability of the newly developed equation and the use of a representative sample of Sri Lankan adolescents with different activities, including a range of free-living activities, is recommended for such validation.

This equation will be useful in observational and randomized control trials to determine the effect of physical activity interventions to promote physical activity in children. Further cross-validation studies are required to assess the validity of this new equation using a representative sample of Sri Lankan adolescents with different activities, including a range of laboratory-based and free-living activities.

## Figures and Tables

**Table 1:** Basic demographic and anthropometric parameters of validation and cross-validation groups by sex.

	Validation Group (*n* = 54)		Cross-Validation Group (*n* = 25)	
Characteristic	Males (*n* = 28)	Females (*n* = 26)	Males (*n* = 13)	Females (*n* = 12)
Age (years)	12.0 ± 0.8	12.0 ± 0.8	12.0 ± 0.8	12.1 ± 0.8
Weight (kg)	34.2 ± 7.5	36.8 ± 9.2	35.6 ± 6.5	33.8 ± 5.7
Height (m)	1.4 ± 0.1	1.5 ± 0.1	1.4 ± 0.1	1.5 ± 0.1
BMI (kg m^−2^)	16.5 ± 2.8	16.9 ± 2.9	17.5 ± 2.4	15.6 ± 1.6
FFM (kg)	25.1 ± 4.3	25.0 ± 5.5	26.2 ± 4.3	24.3 ± 4.2
FM (kg)	9.1 ± 5.2	11.8 ± 5.7	9.4 ± 3.3	9.5 ± 2.5
TEE_DLW_ (kcal/min)	1.38 ± 0.28	1.21 ± 0.29	1.49 ± 0.34	1.22 ± 0.25
PAEE_DLW_ (kcal/min)	0.38 ± 0.24	0.30 ± 0.21	0.46 ± 0.30	0.32 ± 0.21
CPM	403.5 ± 181.7	388.9 ± 163.6	446.9 ± 205.3	415.6 ± 170.8

**Table 2:** Preliminary sex non-specific and sex-specific equations for prediction of PAEE (kcal/min) from the validation group (*n* = 54).

	Prediction Equation	R	R^2^	RMSE
Sex non-specific	PAEE = (0.001CPM) − 0.079	0.81	65.4	0.134
Sex-specific	Male: PAEE = (0.001CPM) − 0.044	0.81	66.1	0.139
	Female: PAEE = (0.001CPM) − 0.111	0.82	67.2	0.122

PAEE, physical activity energy expenditure; CPM, counts per minute; RMSE, root mean squared error.

## Data Availability

Researchers interested in accessing the data are encouraged to contact the research team.

## References

[1] Carson V., Hunter S., Kuzik N., Gray C.E., Poitras V.J., Chaput J.P., Saunders T.J., Katzmarzyk P.T., Okely A.D., Connor G.S. (2016). Systematic review of sedentary behaviour and health indicators in school-aged children and youth: An update. Appl. Physiol. Nutr. Metab..

[2] (2009). Assessment of Body Composition and Total Energy Expenditure in Humans Using Stable Isotope Technique. Vol. 3, Internatonal Atomic Energy Agency Human Health Series. https://www-pub.iaea.org/MTCD/Publications/PDF/Pub1370_web.pdf.

[3] Ekelund U.L., Sjöström M., Yngve A., Poortvliet E., Nilsson A., Froberg K.A., Wedderkopp N., Westerterp K. (2001). Physical activity assessed by activity monitor and doubly labeled water in children. Med. Sci. Sport. Exerc..

[4] Nilsson A., Brage S., Riddoch C., Anderssen S.A., Sardinha L.B., Wedderkopp N., Andersen L.B., Ekelund U. (2008). Comparison of equations for predicting energy expenditure from accelerometer counts in children. Scand. J. Med. Sci. Sport.

[5] Corder K., Brage S., Wright A., Ramachandran A., Snehalatha C., Yamuna A., Wareham N.J., Ekelund U. (2010). Physical activity energy expenditure of adolescents in India. Obesity.

[6] Kuriyan R. (2018). Body composition techniques. Indian J. Med. Res..

[7] Sallis J.F., Saelens B.E. (2000). Assessment of physical activity by self-report: Status, limitations, and future directions. Res. Q. Exerc. Sport.

[8] Ekelund U., Sardinha L.B., Anderssen S.A., Harro M., Franks P.W., Brage S., Cooper A.R., Andersen L.B., Riddoch C., Froberg K. (2004). Associations between objectively assessed physical activity and indicators of body fatness in 9-to 10-y-old European children: A population-based study from 4 distinct regions in Europe (the European Youth Heart Study). Am. J. Clin. Nutr..

[9] Yıldırım M., Verloigne M., De Bourdeaudhuij I., Androutsos O., Manios Y., Felső R., Kovács É., Doessegger A., Bringolf-Isler B., Te Velde S.J. (2011). Study protocol of physical activity and sedentary behaviour measurement among schoolchildren by accelerometry-Cross-sectional survey as part of the ENERGY-project. BMC Public Health.

[10] Elmesmari R., Martin A., Reilly J.J., Paton J.Y. (2018). Comparison of accelerometer measured levels of physical activity and sedentary time between obese and non-obese children and adolescents: A systematic review. BMC Pediatr..

[11] Choi L., Liu Z., Matthews C.E., Buchowski M.S. (2011). Validation of accelerometer wear and nonwear time classification algorithm. Med. Sci. Sport. Exerc..

[12] Jeran S., Steinbrecher A., Pischon T. (2016). Prediction of activity-related energy expenditure using accelerometer-derived physical activity under free-living conditions: A systematic review. Int. J. Obes..

[13] De Bourdeaudhuij I., Van Cauwenberghe E., Spittaels H., Oppert J.M., Rostami C., Brug J., Van Lenthe F., Lobstein T., Maes L. (2011). School-based interventions promoting both physical activity and healthy eating in Europe: A systematic review within the HOPE project. Obes. Rev..

[14] Dabare P.M., Wickramasinghe V.P., Waidyatilaka I., Devi S., Kurpad A.V., Samaranayake D., de Lanerolle-Dias M., Wickremasinghe R., Hill A.P., Lanerolle P. (2021). Validation of accelerometer-based energy expenditure equations using doubly-labelled water technique in 11-13 year-old Sri Lankan children. Sri Lanka J. Child Health.

[15] Jayatissa R., Gunathilaka M., Fernando D. National Nutrition and Micronutrient Survey 2012 Part I: Anaemia among Children Aged 6–59 Months and Nutritional Status of Children and Adults. file:///C:/Users/Windows%20User/Downloads/Sri_Lanka_National_Nutrition_and_Micronutrient_Survey_2012.pdf.

[16] Stewart A., Marfell-Jones M., Olds T., de Ridder H. (2011). International Standards for Anthropometric Assessment-ISAK.

[17] Lohman T.G., Ring K., Pfeiffer K., Camhi S., Arredondo E., Pratt C., Pate R., Webber L.S. (2008). Relationships among fitness, body composition, and physical activity. Med. Sci. Sport Exerc..

[18] Schoeller D.A., Ravussin E.R., Schutz Y.V., Acheson K.J., Baertschi P.E., Jequier E.R. (1986). Energy expenditure by doubly labeled water: Validation in humans and proposed calculation. American Journal of Physiology-Regulatory. Integr. Comp. Physiol..

[19] Weir J.B. (1949). New methods for calculating metabolic rate with special reference to protein metabolism. J. Physiol..

[20] Poehlman E.T., Horton E.S. (1989). The impact of food intake and exercise on energy expenditure. Nutr. Rev..

[21] Schofield W.N. (1985). Predicting BMR, new standards and review of previous work. Human Nutrition. Clin. Nutr..

[22] Crouter S.E., Horton M., Bassett D.R. (2012). Use of a 2-Regression Model for Estimating Energy Expenditure in Children. Med. Sci. Sport Exerc..

[23] Nyström D.C., Pomeroy J., Henriksson P., Forsum E., Ortega F.B., Maddison R., Migueles H., Lof M. (2017). Evaluation of the wrist-worn ActiGraph wGT3x-BT for estimating activity energy expenditure in preschool children. Eur. J. Clin. Nutr..

[24] Migueles J.H., Cadenas-Sanchez C., Ekelund U., Delisle Nyström C., Mora-Gonzalez J., Löf M., Labayen I., Ruiz J.R., Ortega F.B. (2017). Accelerometer data collection and processing criteria to assess physical activity and other outcomes: A systematic review and practical considerations. Sport. Med..

[25] Jemaa H.B., Mankai A., Mahjoub F., Kortobi B., Khlifi S., Draoui J., Minaoui R., Karmous I., Hmad H.B., Slama F.B. (2018). Physical activity level assessed by accelerometer and PAQ-C in Tunisian children. Ann. Nutr. Metab..

[26] Kannan K.S., Raj S.S. (2019). Outlier labeling methods for medical data. Logistics, Supply Chain and Financial Predictive Analytics.

[27] Wickramasinghe V.P., Lamabadusuriya S.P., Cleghorn G.J., Davies P.S.W. (2008). Assessment of body composition in Sri Lankan children: Validation of a bioelectrical impedance prediction equation. Eur. J. Clin. Nutr..

[28] Guo S., Chumlea W.C., Cockram D.B. (1996). Use of Statistical methods to estimate body composition. Am. J. Clin. Nutr..

[29] Bland J.M., Altman D. (1986). Statistical methods for assessing agreement between two methods of clinical measurement. Lancet.

[30] Reilly J.J., Kelly L.A., Montgomery C., Jackson D.M., Slater C., Grant S., Paton J.Y. (2006). Validation of Actigraph accelerometer estimates of total energy expenditure in young children. Int. J. Pediatr. Obes..

[31] Alhassan S., Lyden K., Howe C., Kozey S., Nwaokelemeh O., Freedson P.S. (2012). Accuracy of accelerometer regression models in predicting energy expenditure and METs in children and youth. Pediatric Exerc. Sci..

[32] Welch W.A., Bassett D.R., Freedson P.S., John D., Steeves J.A., Conger S.A., Ceaser T.G., Howe C.A., Sasaki J.E. (2014). Cross-validation of waist-worn GENEA accelerometer cut-points. Med. Sci. Sport. Exerc..

[33] Zhu Z., Chen P., Zhuang J. (2013). Predicting Chinese children and youth’s energy expenditure using ActiGraph accelerometers: A calibration and cross-validation study. Res. Q. Exerc. Sport.

[34] Treuth M., Schmitz K., Catellier D., McMurray R., Murray D., Almeida M., Going S., Norman J.E., Pate R. (2004). Defining accelerometer thresholds for activty intensities in adolescent girls. Med. Sci. Sport. Exerc..

[35] Trost S.G., Ward D.S., Moorehead S.M., Watson P.D., Riner W., Burke J.R. (1998). Validity of the CSA activity monitor in children. Med. Sci. Sport. Exerc..

[36] Puyau M.R., Adolph A.L., Vohra F.A., Butte N.F. (2002). Validation and calibration of physical activity monitors in children. Obes. Res..

[37] Schmitz K.H., Treuth M., Hannan P., Ring K.B., Catellier D., Pate R. (2005). Predicting energy expenditure from accelerometry counts in adolescent girls. Med. Sci. Sports Exerc..

[38] Freedson P., Pober D., Janz K.F. (2005). Calibration of accelerometer output for children. Med. Sci. Sport. Exerc..

[39] Mattocks C., Leary S.A.M., Ness A., Deere K., Saunders J., Tilling K., Kirkby J., Blair S.N., Riddoch C. (2007). Calibration of an accelerometer during free-living activities in children. Int. J. Pediatr. Obes..

[40] Williams J., Townsend N., Rayner M., Jayawardena R., Katulanda P., Manoharan S., Wickramasinghe K. (2019). Diet quality of adolescents in rural Sri Lanka based on the Diet Quality Index–International: Findings from the ‘Integrating Nutrition Promotion and Rural Development’ project. Public Health Nutr..

[41] Waidyatilaka I., de Silva A., de Lanerolle-Dias M., Atukorala S., Lanerolle P. (2016). A field tool for prediction of body fat in Sri Lankan women: Skinfold thickness equation. J. Health Popul. Nutr..

[42] Treuth M.S., Figueroa-Colon R., Hunter G.R., Weinsier R.L., Butte N.F., Goran M.I. (1998). Energy expenditure and physical fitness in overweight vs non-overweight prepubertal girls. Int. J. Obes..

[43] Corder K., Brage S., Ekelund U. (2007). Accelerometers and pedometers: Methodology and clinical application. Curr. Opin. Clin. Nutr. Metab. Care.

[44] Sirard J.R., Pate R.R. (2001). Physical activity assessment in children and adolescents. Sport. Med..

